# ‘Primary small cell carcinoma of tonsil: An extreme rarity.’

**DOI:** 10.1016/j.amsu.2019.06.010

**Published:** 2019-06-21

**Authors:** Rajeev Sen, Namita Bhutani, Reeti Saini, Jashanpreet

**Affiliations:** Deptt. of Pathology, PGIMS Rohtak, Haryana, India

**Keywords:** Immuno histochemistry, Neuroendocrine carcinoma, Small cell carcinoma, Tonsil

## Abstract

**Introduction:**

Small cell neuroendocrine carcinoma (NEC) that originates in the tonsil is extremely rare and carries a poor prognosis. Only a few cases of this tumor have been reported so far and the standard treatment protocol remains uncertain.

**Case report:**

Here, we describe a 65-year-old woman presenting with throat pain. Computed tomography (CT) scan revealed a mass with moderate enhancement in the right tonsil. A biopsy of the tonsillar mass was performed and histologic examination revealed small round to oval tumor cells arranged in cords or nests, containing hyperchromatic nuclei and scant cytoplasm. Mitotic figures were readily identified. Immunohistochemical staining further corroborated the diagnosis. A diagnosis of primary small cell NEC of the left tonsil was rendered. The patient was treated by six cycles of cisplatin combined with etoposide and the masses showed initial complete response. We also provide a succinct review of all tonsillar SCC cases reported in the English literature and their outcomes.

**Conclusion:**

Small Cell Carcinoma of the tonsil is an extremely rare entity with an aggressive disease course and poor prognosis. Therefore, it is important for the clinicians to be aware of the uncommon occurrence of this disease and its management.

## Highlights

• Small cell NECs of the tonsil are extreme­ly rare and only a few cases were added since it was firstly repoted.
• The cell of origin was initially thought to be from amine-precursor uptake and decarboxylase (APUD or Kulchitsky) cells; however, the current opinion favors their origin from multipotential stem cells.
• Thus far, the therapeutic strategy has not been properly formulated due to the paucity of data.

## 1 Introduction

Tonsil is one of the most common sites from where primary oropharyngeal tumors arise. The most common tumor in this area is squamous cell carcinoma, while minor salivary gland tumors, lymphomas, melanoma and sarcomas are also included [1]. Small cell neuroendocrine carcinomas are poorly differentiated tumors. Although bronchogenic SCC is most common, origin from gastrointestinal, genitourinary, breast, head and neck and unknown primary have been reported. In head and neck region, the incidence of primary small cell NEC is low. Larynx is the relative most commonly involved site, followed by salivary glands, nasal cavity and paranasal sinuses [2]. Small cell NECs of the tonsil are extremely rare and only a few cases were added since it was firstly reported by Koss et al. in 1972 [3]. According to a recent review by Renner [2], extrapulmonary SCC constitutes about 2.5–5% of all SCCs [4]. The cell of origin in the head and neck SCC was initially thought to be from amine-precursor uptake and decarboxylase (APUD or Kulchitsky) cells; however, the current opinion favors their origin from multipotential stem cells [[3], [4], [5]].Thus far, the therapeutic strategy has not been properly formulated due to the paucity of data. Here we present a rare case of primary small cell NEC arising from the tonsil and the clinical and pathological features of this case were described. It displays aggressive biologic behavior and is associated with relatively poor prognosis.

## 2 Case report

**Fig. 1:**
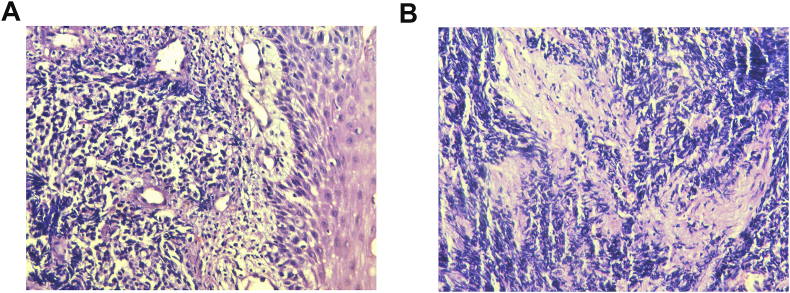
**A**: Small round to oval tumor cells arranged in cords or nests. (H&E 100X), **B**: Tumor cells arranged in cords or nests, containing hyperchromatic nuclei and scant cytoplasm, nuclear molding, numerous mitotic FIGURES AND apoptotic bodies. (H&E 200X).

**Fig. 2:**
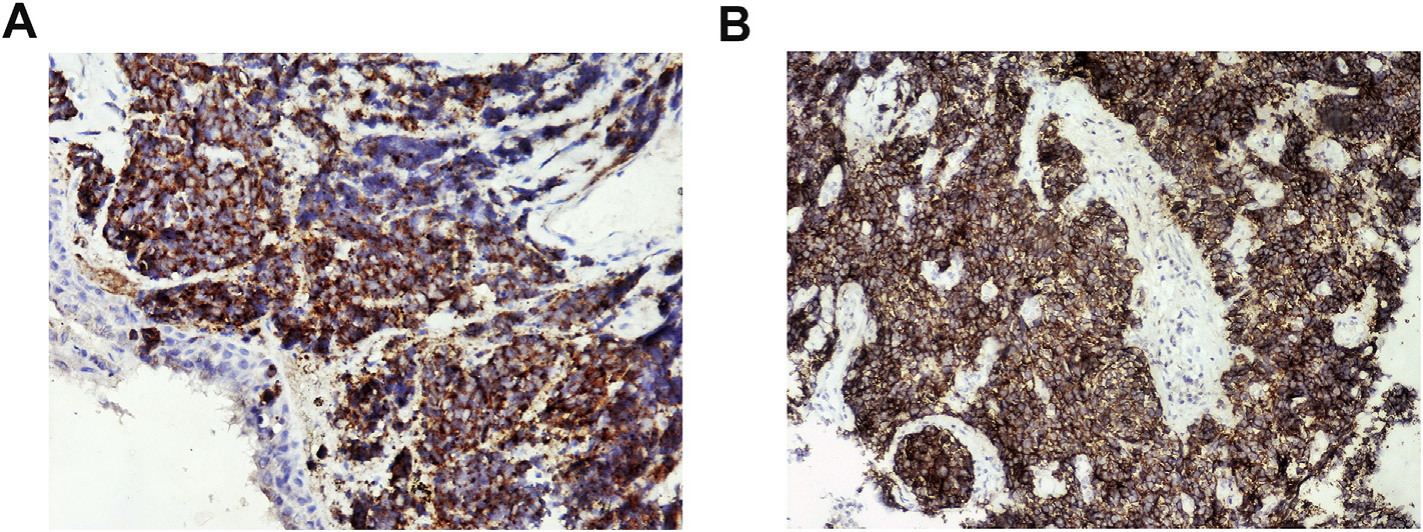
**A**: SYNAPTOPHYSIN POSITIVE IN TUMOR CELLS (200X). **b**: CD56 POSITIVITY IN TUMOR CELLS. (200X).

A 65 year-old female was referred to ENT department with complaint of throat pain for one month. There was no history of smoking and alcohol consumption. History of dysphagia or dyspnea was denied but odynophagia was present. The initial treatment with antibiotics was administered by her primary care physician but was not effective. Physical examination revealed an enlarged right palatine tonsil with ulcerating mucosa and fullness of her anterior tonsillar pillar. The left palatine tonsil and other pharyngeal mucosal surfaces were normal. The remainder examination of head and neck was negative. A computed tomography (CT) scan of the neck showed a 3.5 × 2.8 cm well-circumscribed, enhancing, necrotic appearing mass posterior to the right submandibular gland and increased attenuation within the right tonsil. A CT scan of the chest, abdomen and pelvis was unremarkable. Whole body positron emission tomography – computed tomography (PET-CT) showed a prominent collection of fluorodeoxyglucose within right tonsillar fossa without any evidence of distant metastatic disease. Fine-needle aspiration of the right neck mass performed at an outside hospital was positive for poorly differentiated carcinoma. On endoscopy, a very small exophytic tumor involving the right palatine tonsil was seen. Several biopsies were taken and sent for histology. Histologic examination revealed small round to oval tumor cells arranged in cords or nests, containing hyperchromatic nuclei and scant cytoplasm, nuclear molding, numerous mitotic figures and apoptotic bodies (Fig. 1A and B). Immunohistochemical staining showed that tumor cells were strongly positive for neural cell adhesion molecule (CD56), Synaptophysin, Chromogranin, NSE and negative for leukocyte common antigen (LCA) and CD 20 (Fig. 2A and B). The patient was diagnosed with poorly differentiated neuroendocrine carcinoma (SCC) of the right tonsil. Thus the right palatine tonsil was confirmed as the primary lesion and there was no evidence of distant metastasis. As she refused surgery or radiotherapy, six cycles of Cisplatin combined with Etoposide were given and the mass showed initial complete response at that time. The patient refused any further treatment and was lost to follow-up.

## 3 Discussion

Small cell NECs are most commonly seen in lungs and have been reported to occur in some extra-pulmonary sites, mainly in alimentary and genitourinary system [6] A limited number of reports have described small cell NECs arising from the head and neck region and shows the relative propensity for larynx, followed by salivary glands and sinonasal region [2]. Small cell NEC that primarily occurs in tonsil is extremely rare. Tonsillar Small cell carcinoma (SCC) occurs most often in the 5th to 7th decades, and is more common in males than females (2:1 ratio). The tumor commonly presents clinically as a painless neck mass. Although paraneoplastic syndromes including SIADH, Cushing's syndrome and Eaton-Lambert myasthenic syndrome have been associated with head and neck SCC, there are no reports of these syndromes being associated with tonsillar SCC [2].

On light microscopy, hallmarks of small cell NEC include small round to oval cells packed in sheets, cords or ribbons with hyperchromatic nuclei, sparse cytoplasm, high nuclear/cytoplasmic ratio and frequent necrosis and mitosis. On immunohistochemistry, positive staining of general neuroendocrine markers including synaptophysin, chromogranin, NSE and CD56 can provide evidences of neuroendocrine differentiation of tumor cells.

A large number of terms have been used when referred to small cell NEC. Synonyms of this tumor include small cell carcinoma, oat cell carcinoma, anaplastic small cell carcinoma and poorly differentiated (grade III) neuroendocrine carcinoma. Three cases of small cell NEC of the tonsil were initially reported by Koss et al. in 1972 [3]. During the past 40 years, there have been only 14 cases added in the English literature [[7], [8], [9], [10], [11], [12], [13], [14]]. Two cases were excluded due to lack of powerful evidence of primary tonsillar origin or adequate data in pathology [8,12]. Thus, 12 of these cases, with sufficient clinicopathologic data, are summarized in Table 1.

**Table 1:** Clinicopathologic data of the reported cases.

LITERATURE	CASE NO.	AGE/SEX	INITIAL SYMPTOMS	TUMOR SITE & SIZE	TREATMENT	RESULTS
KOSS ET AL (1972)	1 2 3	70/F 60/M 54/M	N.G. N.G. N.G.	TONSIL (5 CM) TONSIL(OCCULT) TONSIL (3.5 CM)	RT RND+RT LOCAL EXCISION+RND	DOD 8 MOS DOD 18 MOS DOD 1.5 YRS
ABEDL & SISMANIS (1987)	4	67/F	LEFT NECK MASS	LEFT TONSIL (N.G.)	RT+CT	DOD 6 MOS
HEIMANN ET AL (1995)	5	78/N.G.	LEFT NECK MASS	LEFT TONSIL (N.G.)	LEFT RND	DEATH DUE TO CARDIAC ARREST
BAWA & WAX (1995)	6	53/M	ODYNOPHAGIA	LEFT TONSIL (N.G.)	CT+RT	DOD 15 MOS
WENG ET AL (2008)	7	53/M	RIGHT NECK MASS	RIGHT TONSIL (N.G.)	CT+RT	DOD 6 MOS
HATOUM ET AL (2009)	8 9 10	49/M 50/M 50/F	N.G. N.G, N.G.	T1N3 T3N1 T4N2b	CRT CRT C RT	DOD 2.5 YRS ALIVE ALIVE
SEGAWA ET AL (2011)	11	65/F	SORE THROAT & LEFT NECK MASS	LEFT TONSIL (3.0 CM)	RT+CT	DOD 2 YRS
SEHDEV ET AL (2012)	12	53/M	RIGHT NECK MASS	RIGHT TONSIL (N.G.)	CRT	ALIVE

Abbreviations: yrs: years; F: female; M: male; N.G.: not given; RT: radiotherapy; DOD: die of disease; mos: months; MET: metastases; RND: radical neck dissection; CT: chemotherapy; REC: recurrence; CRT: chemoradiotherapy; NED: no evidence of disease.

In our review, most small cell NECs of the tonsil occurred in patients between 50 and 66 years of age (range, 49–78 years), with the male/female ratio 1.75: 1. Of the patients reported to date, cardinal symptom of this tumor was neck mass. The rapid clinical course (from 2 weeks to 3 months) and progressive enlargement of the neck mass may suggest the malignant behavior of this tumor. Notably, in all of the 9 cases (locations of neck nodes were not given in 3 cases), cervical metastatic lymphadenopathy were from the ipsilateral primary lesions. Nevertheless, we failed to extract further information about the regularity of the neck sublevel involvement. Other symptoms included throat pain and odynophagia which were also presented in our case.

Although apparent lesions in tonsil as asymmetric swelling and ulcerating mucosa can be well recognized by physical examination, small submucosal tumors (case No. 2, for instance) might be missed without radiographic evaluation. Fine-cut imaging with CT and/or MRI can evaluate the tumor size and its infiltration tumor is not metastasis of other distant primary, especially from the lung [15]. The diagnosis of small cell NEC is based on its pathological and immunohistochemical features which have been mentioned above. Differential diagnosis includes paraganglioma (which is positive staining for S-100 but negative for cytokeratin) and malignant lymphoma (which is immunoreactive for LCA but negative for neuroendocrine markers) [16].

Besides the locoregional spread to cervical lymph nodes, the tumor metastasizes most commonly to liver, lungs, bones, brain and skin [17]. Owing to its rarity, recommendations for management of small cell NEC of the tonsil have not been established. In general, it is wildly accepted that small cell NEC of the head and neck is aggressive and prone to develop early regional or distant metastasis. Based on comparative treatment for small cell NEC of the larynx and lung, various modalities including surgical resection, radiotherapy, chemotherapy or some combination of them have been indicated. In terms of local control, current opinion favors the use of radiotherapy to the primary tumor site and neck rather than surgery, or their combination, on account of their indistinctive outcome [2]. With limited controversy, many investigators suggested that chemotherapy should be considered in all patients with small cell NEC of the head and neck by reason of its propensity for early metastasis. Among all the chemotherapeutic agents, platinum-based regimens such as CDDP and etoposide are most commonly used in recent years. Use of new chemotherapeutic agents such as irinotecan, which has shown encouraging effect against small cell lung cancer, has also been reported in one case of small cell NEC arising from the tonsil [13]. Despite multimodality treatment, the outcome of patients with small cell NEC of the head and neck is dismal. By our review of the 12 cases, recurrence or distant metastases was found in 66.7% of them and these patients ultimately died of disease in 2.5 years with the median overall survival time of 18.0 months. The sites of distant metastases were the lung, liver, bone and one unusual location, the adrenal.

## 4 Conclusion

Small Cell Carcinoma of the tonsil is an extremely rare entity with an aggressive disease course and poor prognosis. Therefore, it is important for the clinicians to be aware of the uncommon occurrence of this disease and its management. With a paucity of studies, standard treatment protocol remains uncertain while radiotherapy combined with chemotherapy seems to be the relative appropriate option. Further improvements in our understanding of the pathology and treatment strategies are needed to combat this disease.

We state that the work has been reported in line with the SCARE criteria [18]. The due consent has been taken from the patient for possible publication of this case report.

## Ethical approval

Institutional review board approval was not required because all data were collected from clinical records.

## Consent

Written informed consent was obtained from the patient for publication of this case report and accompanying images. A copy of the written consent is available for review by the Editor-in-Chief of this journal on request.

## Sources of funding

Author declare there is no funding resources for this paper.

## Author contribution

DR. NAMITA BHUTANI WROTE THE PAPER.

DR RAJEEV SEN: REPORTED THE CASE.

DR. JASHANPREET: READ THE PAPER.

DR REETI SAINI: ANALYSED THE PAPER.

## Registration of research studies

NOT APPLICABLE.

## Conflicts of interest

The authors declare that there is no conflict of interest regarding the publication of this paper.

## Trial registry number

None.

## Guarantor

DR. NAMITA BHUTANI the corresponding author of this paper.

## Provenance and peer review

Not commissioned, externally peer reviewed.
